# A Decision Support Method for Prehospital Emergency Care Based on Ranking the Importance of Physiological Variables

**DOI:** 10.3390/healthcare8030295

**Published:** 2020-08-24

**Authors:** Li Zhang, Shuying Zhao, Fang Li, Guozheng Rao

**Affiliations:** 1 School of Economics and Management, Tianjin University of Science and Technology, Tianjin 300457, China; zhangli2006@tust.edu.cn; 2 College of Intelligence and Computing, Tianjin University, Tianjin 300350, China; m18322302639@163.com; 3 School of Biomedical Informatics, University of Texas Health Science Center at Houston, 7000 Fannin St Suite 600, Houston, TX 77030, USA; Fang.Li@uth.tmc.edu; 4 Tianjin Key Laboratory of Cognitive Computing and Applications, Tianjin University, Tianjin 300350, China

**Keywords:** machine learning, physiological variable, disease, ICU

## Abstract

To the on-site nursing staff or field management in prehospital emergency care, it seems baffling to conduct more targeted checklist tests for a specific disease. To address this problem, we proposed a decision support method for prehospital emergency care based on ranking the importance of physiological variables. We used multiple logistic regression models to explore the effects of various physiological variables on diseases based on the area under the curve (AUC) value. We implemented the method on the intensive care database (i.e., the Medical Information Mart for Intensive Care (MIMIC-III) database) and explored the importance of 17 physiological variables for 24 diseases, both chronic and acute. We included 33,798 adult patients, using the full physiological dataset as experiment data. We ranked the importance of the physiological variables related to the diseases according to the experiments’ AUC value. We discussed which physiological variables should be considered more important in adult intensive care units (ICUs) for prehospital emergency care conditions. We also discussed the relationships among the diseases based on ranking the importance of physiological variables. We used large-scale ICU patient data to obtain a cohort of physiological variables related to specific diseases. Ranking a cohort of physiological variables is a cost-effective means of reducing morbidity and mortality under prehospital emergency care conditions.

## 1. Introduction

Improving the quality and performance of prehospital emergency care (PEC) is a cost-effective way of reducing morbidity and mortality under emergency conditions [1,2]. Some studies are beginning to investigate the feasibility of checklists in improving prehospital emergency care [3,4]. For instance, Kang et al. proposed an artificial intelligence (AI) algorithm based on deep learning to predict the severity of a patient’s medical condition in emergency medical services (EMSs) [5]. However, obtaining the checklists for emergency conditions using AI is still a big problem.

To address this problem, data-driven analytical approaches played an increasingly important role in clinical practice and research in the application of the rapidly developing medical database. As such, there are opportunities to transform healthcare using “big clinical data” [6]. Using clinical data to explore the relationships between physiological variables (PVs) and diseases was an essential topic in clinical research. For diseases in intensive care units (ICUs), it is necessary to understand the importance of PVs in the disease process. For PEC, our work can help to more comprehensively understand the PVs related to diseases. Then, a checklist can be constructed during PEC using our method. Moreover, based on the influence of PVs on a disease, associations among diseases can be found, promoting the disease research process.

Many machine learning studies have been carried out in recent years using clinical databases. Most research has focused on the application of the electronic health record (EHR), which can be used to predict discharge medications at admission time based on a convolutional neural network (CNN) model [7]. Of course, there are also a few studies aimed at PVs and diseases, for example, using 13 frequently employed clinical measurements based on long short-term memory networks (LSTMs) to classify diagnoses in pediatric intensive care unit (PICU) [8]. However, research in this field is scarce, even though there are many records in clinical data, including electronic health records, disease diagnoses, and the monitoring of PVs. PVs are temporal series representing the evolution of a physical variable related to a patient’s physio-pathological state. Generally, a disease is related to multiple PVs. In addition to the lack of research in this field, there are few methods to explore the knowledge gap between multiple PVs and diseases.

In ICUs, the useful analysis of the effects of multiple PVs on a disease can help to provide better care for patients with a specific disease and to promote the disease research process. In medical data, PVs are well documented and reflect the evolution of a patient’s condition. Therefore, among the many PVs recorded, a comprehensive understanding of the importance of each PV to a disease can improve the quality of care. Moreover, if different diseases are associated with multiple identical PVs, it will provide a point of contact for disease research. Both obesity and hypertension are closely related to body weight, and obese people often have high blood pressure. Therefore, based on the relationship between PVs and diseases, PVs may be a bridge in the study of disease relationships (i.e., subtypes of the disease and complications).

The main contributions of this paper are summarized below. First, we proposed a decision support method for prehospital emergency care based on ranking the importance of PVs. The most important multiple PVs precisely reflect the conditions of patients and differ from the results provided by a single PV for a disease. We proposed a method using machine learning to explore the relationship between multiple PVs and a specific disease using large-scale data. We found that in ICUs, a cohort of PVs related to a specific disease is sufficient to understand the disease and take care of the patient. Then, we proposed PVs that should be given the most attention in adult ICUs for common conditions, including critical diseases and chronic diseases. In real PEC, the ranked PVs can be referenced as a potential PV checklist for a specific disease. It also can offer easy-to-follow instructions, reduce diagnostic and therapeutic errors, and change incorrect behaviors. Finally, we were able to find links between the sets of two diseases based on PVs. For example, the PVs ranked results show that the top three PVs are the same between ‘conduction disorder’ and ‘coronary atherosclerosis and other heart diseases’. It is verified that conduction disorder is usually a complication of coronary atherosclerosis and other heart diseases. Another example shows that the second and third PVs are different between ‘diabetes mellitus with complications’ and ‘diabetes mellitus without complications.’ It is known that diabetes mellitus with complications and diabetes mellitus without complications are different subtypes of the same disease. Therefore, more important PVs, including PV(Glucose), should be monitored to distinguish different subtypes of diabetes mellitus.

## 2. Related Work

The burden on EMSs has increased considerably in many countries [9]. For example, in the last ten years, prehospital EMS demand and emergency response times in Beijing have increased [10], and therefore, more resources and effort should be devoted to PEC. The Danish prehospital EMS care system has received major changes [11], and other national organizations have identified a need to incorporate more evidence-based medicine in PEC [12]. For example, physician-manned prehospital EMSs have become widespread in Japan, leading to improved outcomes for patients who are likely to require critical care in the prehospital phase [13].

The quality and performance of PEC are essential for saving more people in EMSs [1,2]. For instance, the PEC systems in the defense medical services have been proven to save lives [14]. Indeed, PEC enables the patient to be transported on the right platform, with the right medical team, to the right place. In Swedish PEC, the majority of adverse events originate as a result of deviations from the normal standard of care and incomplete documentation [15]. In EMSs, accurately predicting the severity of a patient’s medical condition with an AI algorithm could overcome the limitations of conventional statistical methods and have recently achieved state-of-the-art performance [5]. Decisions made by paramedics during PEC have the potential to impact patient health outcomes and represent both a professional risk for individual staff members, and a reputational risk to with regards to patient trust [16]. In PEC, clinical decision support systems are not designed to replace humans, but can help healthcare providers to remember relevant diagnostics and checklists (i.e., treatment options) to improve the quality of PEC [17]. The checklists for PEC may help to improve adherence to treatment guidelines [3], although the optimal PEC management is still under study [12]. For example, EMS care for acute stroke varies widely across the United States; however, EMSs need more uniformed high-quality care and specific standards for evaluating PEC [4]. Historically, performance within the PEC setting has been assessed primarily based on response times. For instance, in South Africa, more PEC-specific quality indicators, including response time, have improved PEC performance [18].

There is an extensive body of research on using the clinical database. Still, most studies have explored the relationship between a single indicator and a single disease using small-scale data. In medical research, an increasing number of studies are exploring the relationships between PVs and diseases [19]. For example, the clinical control method has been applied to explore the relationship between acute myocardial infarction (AMI) and glucose [20]. Meanwhile, Isabel et al. [21] found an abnormal partial discharge of electroencephalography in Parkinson’s patients. The relationship between the glycemic index and diseases, such as obesity, diabetes mellitus, and cardiovascular disease, were explored in the works of Vega-López and Mårtensson et al. [22,23]. Many factors have been shown to be associated with renal failure, such as high human body temperature and hypocomplementemia. The results provided by DeQuattro et al. [24] suggest that there is a close relationship between human body temperature and renal failure. Additionally, in the work of Tumer et al. [25], it was found that pH levels could affect the probability of perioperative complications (e.g., cardiac surgery-associated acute kidney injury).

Machine learning methods have been used to explore relationships between PVs and diseases. In the work of Fan et al. [26], a constraint-based causality discovery method was proposed to obtain more stable causal relationships. In the work of Jin et al. [27], the random forest (RF), linear discriminant analysis (LDA), and support vector machine (SVM) methods were used to explore the relationship between PVs (i.e., airflow and oxygen saturation signals) and chronic obstructive pulmonary disease.

## 3. Methods

**Figure 1:**
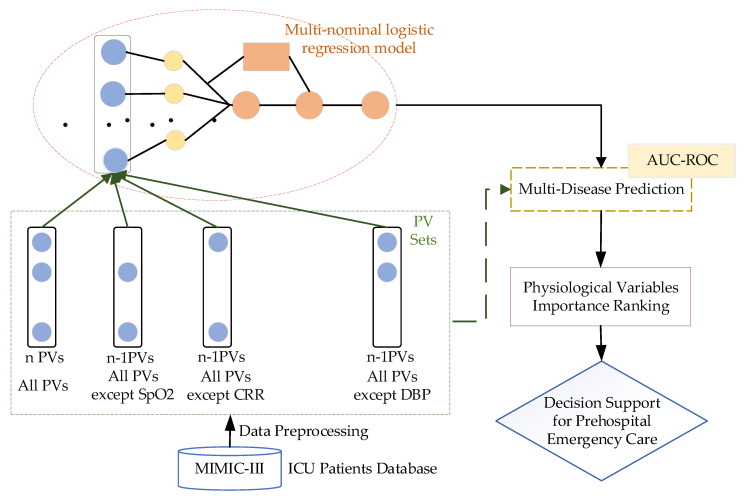
A decision support method for prehospital emergency care based on ranking the importance of physiological variables (PVs). AUC-ROC, area under the receiver operator characteristic curve; ICU, intensive care unit; MIMIC-III, Medical Information Mart for Intensive Care; SpO2, oxygen saturation; CRR, capillary refill rate; DPB, diastolic blood pressure.

Most previous studies have focused on the relationship between a single indicator and a single disease, and most were based on small datasets (i.e., hundreds of patients). Here, we present a decision support method for PEC based on ranking the importance of PVs. Our research aims to explore the relationships between multiple PVs and a specific disease using large-scale clinical data. As shown in Figure 1, the architecture of our method includes the ICU patients database and data preprocessing, PV set design, and multi-nominal logistic regression models [28].

### 3.1. ICU Patient Database and Data Preprocessing

#### 3.1.1. ICU Database and Data Preprocessing

**Figure 2:**
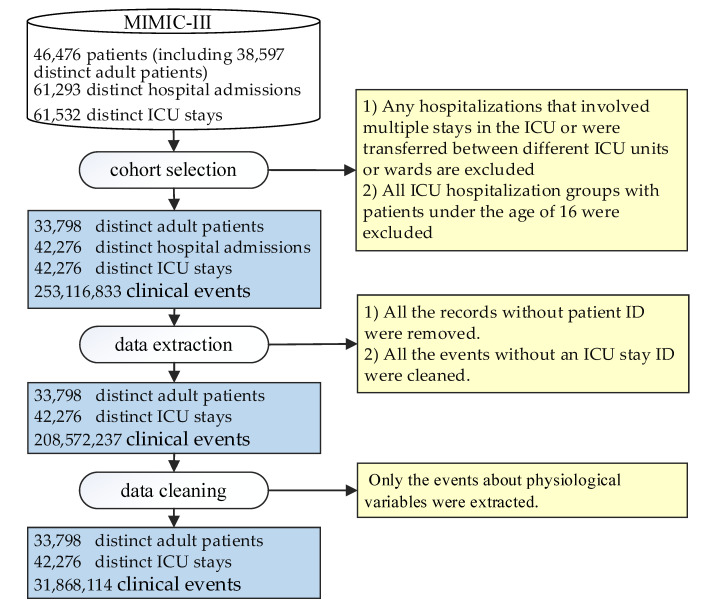
The cohort selection, data extraction, and data-cleaning process of the experimental dataset.

In Figure 2, our study data were derived from MIMIC-III [29], a real-world database. It is a publicly available critical care database, and online courses and tests must be passed to gain access. MIMIC-III contains data associated with 53,423 distinct hospital admissions for adult patients (aged 16 years or above) admitted to critical care units between 2001 and 2012, as well as 7870 neonates admitted between 2001 and 2008; in total, it contains data associated with 61,293 distinct hospital admissions, covering 38,597 distinct adult patients and 49,785 hospital admissions. Given the data richness and authenticity of MIMIC-III, we chose it as our data source for this research. One of the significant challenges facing machine learning in medicine is the lack of recognized benchmarks. Previous studies have proposed a public benchmark for four different clinical tasks, supported by the MIMIC-III database [30]. Therefore, our research inherits some ideas from this benchmark.

#### 3.1.2. Cohort Selection

First, due to the physiological differences between children and adults, we selected patients who were over 16 years at the time of ICU admission. Second, each patient had one or more admission records for one or more hospitals. During a single admission, as the patient’s condition changes, the patient may be hospitalized one or more times and may even enter the ICU. The clinical events recorded on each admission are individual measurements, observations, or treatments. With the development of the patient’s disease, patients may be transferred from one department to another, or even from one hospital to another, and the patient’s medical information records will be discontinuous during the transfer. Thus, we only used their single admissions without complex and transferred information for each patient to prevent possible information interference in the analysis.

#### 3.1.3. Data Extraction

MIMIC-III records a large amount of medical data from patients. These data include the entire diagnosis and treatment process of patients from admission to discharge, and the data types include text records and digital medical data. In our study, we chose to focus on the PV monitoring data and disease diagnosis record. Therefore, we identified the table that recorded the relevant information and then selected the required data. These tables were: ADMISSIONS; PATIENTS; ICUSTAYS; D_ICD_DIAGNOSES; DIAGNOSES_ICD; CHARTEVENTS; LAB EVENTS; and OUTPUTEVENTS.

In the selection process of PVs and diseases, as there are many complicated diseases in the records, we focused on the impact of PVs on the 24 diseases shown in Table 1. These 24 diseases included acute illnesses, chronic diseases, and some diseases with periodic attacks. In the raw spreadsheets, there was a series of measurements taken in the emergency department throughout the patient’s hospital admissions. There were upwards of hundreds of surveillance records, and more than 40 in the Physionet/CinC Challenge 2012 alone [31]. We chose 17 essential PVs because these are ubiquitous in each patient in the ICU and have extensive research applicability. The details of each PV are provided in Table 2.

#### 3.1.4. Data Cleaning

To obtain the final experimental data, we cleaned the data, including the correction and deletion of some values. Data extracted from the MIMIC-III database have many erroneous entries, including noise, missing values, outliers, and measurement unit errors. During this step, we obtained the records of PVs and diseases, and the resulting data included around 30,000,000 events.

### 3.2. PV Set Design

Sensitivity analysis is a method of studying and analyzing the sensitivity of changes in the state or output of a system (or model) to changes in the system parameters or surrounding conditions. Sensitivity analysis can determine which parameters have a more significant impact on the system or model and is a guide for evaluating scientific models in practice [32]. Inspired by sensitivity analysis methods, we explored which PVs have a more significant impact on diseases.

If there are *n* PVs considered, we will design *n* + 1 PV sets. The first one (all PV sets) includes all PVs. All PVs except the SpO2 set include all PVs other than SpO2. We obtain *n* − 1 PVs sets with *n* − 1 PVs in the same way. The PV sets are input for multi-disease predictions based on the multinomial logistic regression model. For specific diseases, different AUC values can be obtained for various PV set inputs. Based on these values, we ranked the importance of the PVs for the disease.

### 3.3. Multinomial Logistic Regression Model

**Figure 3:**
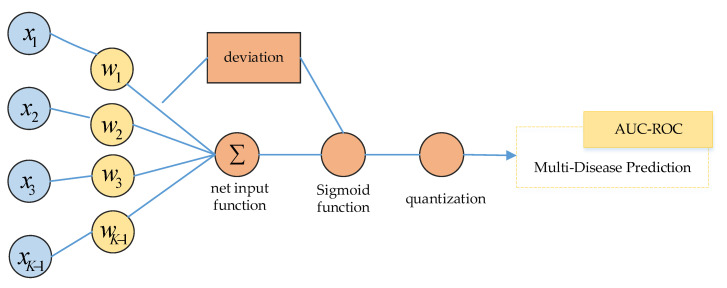
Multinomial logistic regression model.

In Figure 3, multinomial logistic regression is a classification model expressed by conditional probability distribution P(Y|X). Assuming that the range of the discrete random variable Y is {1,2, …, K}, the multinomial logistic regression model is:

(1) $$P\left(Y=k|x\right)=\frac{\exp\left(w_{k}\cdot x\right)}{1+\sum_{k=1}^{K-1}\exp\left(w_{k}\cdot x\right)}$$

(2) $$P\left(Y=K|x\right)=\frac{1}{1+\sum_{k=1}^{K-1}\exp\left(w_{k}\cdot x\right)}$$

where $x\in R^{n+1},\;w_{k}\in R^{n+1},\;k=1,2,\ldots ,K-1$.

#### 3.3.1. Parameter Estimation

Maximum likelihood estimation (MLE) can be used to estimate the model parameters when training the multinomial logistic regression model. In this way, the problem becomes an optimization problem with the logarithmic likelihood function as the objective function:

(3)  P(Y=k|x)=π(x)

(4)  P(Y=K|x)=1−π(x)

Likelihood function:

(5) $$\overset{N}{\underset{i=1}{\prod }}{\left[\pi \left(x_{i}\right)\right]}^{y_{i}}{\left[1-\pi \left(x_{i}\right)\right]}^{1-y_{i}}$$

Logarithmic likelihood function:

(6) $$L\left(w\right)=\sum_{i=1}^{N}\left[y_{i}\left(w\cdot x_{i}\right)-\log\left(1+\exp\left(w\cdot x_{i}\right)\right)\right]$$

We found the maximum value of L(w) and obtained the estimated value of w.

#### 3.3.2. Feature Extraction

**Figure 4:**
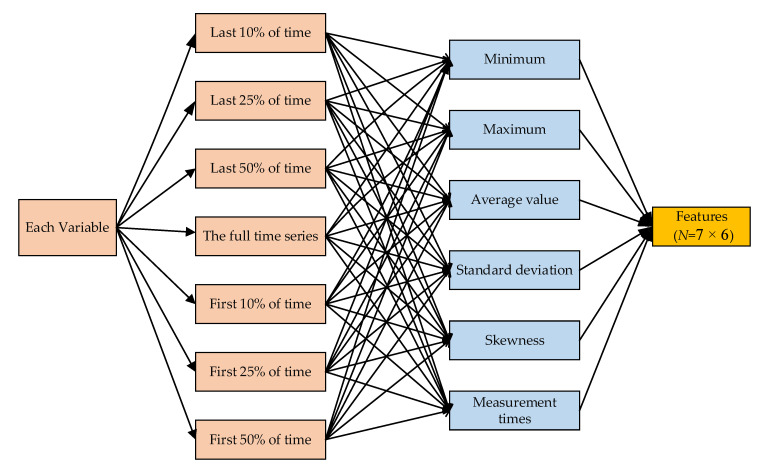
Feature extraction method in multinomial logistic regression.

The details of feature extraction are shown in Figure 4. In this step, we inherited the hand-engineered features of each physiological value presented in the work of Harutyunyan et al. [30]. To enhance the data, we computed six different sample statistic features on seven distinct sub-sequences of a given time series. First, a given time series of a PV was used to calculate seven different features, which were sub-sequences of the full time series: the first 10% of the time, the first 25% of the time, the first 50% of the time, the last 50% of the time, the last 25% of the time, and last 10% of the time. Second, each sub-sequence was used to compute six different sample statistic features, which included the minimum, maximum, mean, standard deviation, skewness, and number of measurements. Finally, each PV value was processed to obtain 42 (7 × 6) features.

## 4. Experiment and Results

### 4.1. Experimental Dataset

In the classification and extraction of patients, it is necessary to extract relevant data from the original MIMIC-III tables and to organize them by patients. The experimental dataset was processed as shown in Figure 2. There were 46,476 patients (including 38,597 distinct adult patients), 61,293 distinct hospital admissions, and 61,532 distinct ICU stays in MIMIC-III. First, during the cohort selection, data with any patient hospitalizations that involved multiple stays in the ICU or transfers between different ICU units or wards were excluded in order to reduce the ambiguity of the results related to hospitalization rather than ICU hospitalization. Moreover, due to the significant differences between adult and pediatric physiology, all ICU hospitalization groups with patients under the age of 16 were excluded, and all records without a patient identifier were removed. During data extraction and cleaning, only the events related to PVs were extracted, and events without an ICU stay identifier were removed. A final total of 33,798 patients were selected, involving 42,276 ICU stays and more than 31 million clinical events.

### 4.2. Experimental Design

For the 24 diseases, we determined which PVs were most important for disease prediction based on the idea of phenotypic classification. We performed 18 experiments based on a polynomial logistic regression model, and we designed 18 PV sets as the input for multi-disease prediction based on a multinomial logistic regression model. The all PVs experiment with 17 PVs was the essential reference experiment for each disease. In each subsequent test, we retained 16 PVs and excluded the other PV. Finally, we obtained AUC values for each disease. For specific diseases, different AUC values can be obtained under various combinations of PVs. Based on these values, we obtained a cohort of PVs that were the strongest predictors of disease in the ICU.

If the AUC value of the disease is significantly reduced because of the absence of a PV in one experiment, the disease is assumed to be significantly associated with this PV, and therefore, more attention should be paid to this PV for patients with this disease. Moreover, if two diseases are associated with the same PVs, there may be a close link between the two diseases—for example, similar diseases, complications, and different subtypes of the same disease.

### 4.3. Results

In the experiments, we obtained a series of AUC values. According to our original records, we got a cohort of PVs related to different diseases. To validate our experimental results, we compared the results with existing knowledge. For example, pH, temperature, and glucose were the top three PVs for acute and unspecified renal failure, in accordance with previous studies [24]. Acute myocardial infarction is also related to glucose [20], and glucose was ranked highly for acute myocardial infarction, stroke, and heart failure. In Table 3, examples of ranking the importance of PVs are shown. Based on existing knowledge, we know that high blood pressure is closely related to blood pressure, and diabetes is closely related to glucose, and these relationships were reflected in our experimental results (shown in Table 3). In summary, for specific diseases in ICUs, we should focus on the top-ranked PVs for each disease.

**Figure 5:**
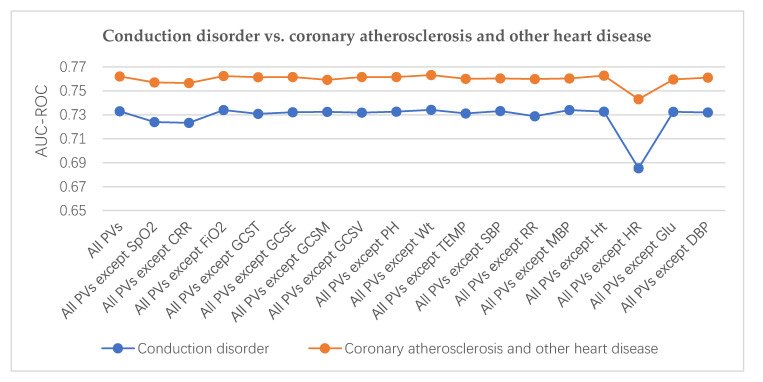
Ranking the importance of PVs (conduction disorder vs. coronary atherosclerosis and other heart diseases).

**Figure 6:**
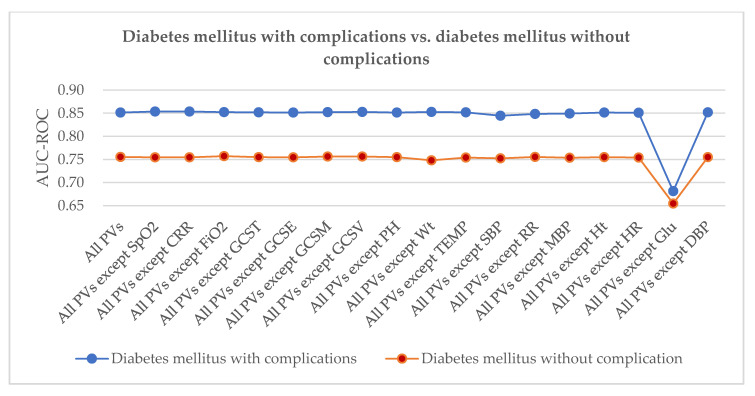
Ranking the importance of PVs (diabetes mellitus with complications vs. diabetes mellitus without complications).

In our method, we found that two different diseases may be related to the same PV. For example, Figure 5 shows that conduction disorder, coronary atherosclerosis, and other heart diseases are linked by the same three PVs—oxygen saturation, capillary filling rate, and heart rate. This suggests a potential link between the diseases. As per confirmation, conduction disorders are usually the clinical complications of coronary atherosclerosis and other heart diseases [33]. Figure 6 shows that diabetes mellitus with complications is closely related to diabetes mellitus without complications. Some top-ranked PVs are important predictors for them—such as glucose, systolic blood pressure, and mean blood pressure. However, the second and third PVs are different between them. It is known that diabetes mellitus with complications and diabetes mellitus without complications are different subtypes of the same disease. Thus, we should monitor more important PVs including PV(Glucose) to distinguish different subtypes of diabetes mellitus.

The results are extensive, and since full validation was not possible, we validated only a subset of the results, which proved that our method can adequately reflect the effects of multiple PVs on specific diseases in ICUs, despite only using a subset. Moreover, correlations between diseases can be captured based on PVs.

## 5. Discussion

The AUC values for coronary atherosclerosis and other heart disease predictions were higher than the AUC values for conduction disorder predictions with the same PV set. This indicates that coronary atherosclerosis and other heart diseases are more sensitive to PVs. Heart rate (HR) was the most sensitive PV for both conditions [33]. However, the AUC values of the conduction disorder prediction decreased more than for coronary atherosclerosis, meaning that the conduction disorder is more sensitive to HR. Similarly, in Figure 6, the AUC values for the diabetes mellitus with predicted complications were all higher than those for diabetes mellitus without predicted complications with the same PV set, indicating that diabetes mellitus with complications is more sensitive to PVs. Glucose was the most sensitive PV for them both. However, the AUC values of diabetes mellitus with complications prediction decreased more than that of diabetes mellitus without complications. Diabetes mellitus with complications is a more serious and a worse condition than diabetes mellitus without complications. In Table 3, systolic blood pressure (SBP) is the second most important PV predictor of diabetes mellitus with complications, which was verified based on hypertension being the most common complication of diabetes mellitus [20].

In practice, the most important PVs that predict various diseases should be monitored. In PEC, the more important PVs of ICU patients should be tested with skilled training. The ranked PVs can be referenced as a potential PVs checklist for a specific disease, offering easy-to-follow instructions, reducing diagnostic and therapeutic errors, and altering incorrect behaviors. This can help to improve the quality of PEC and to save more patients.

### 5.1. Comparison to Other Methods

**Figure 7:**
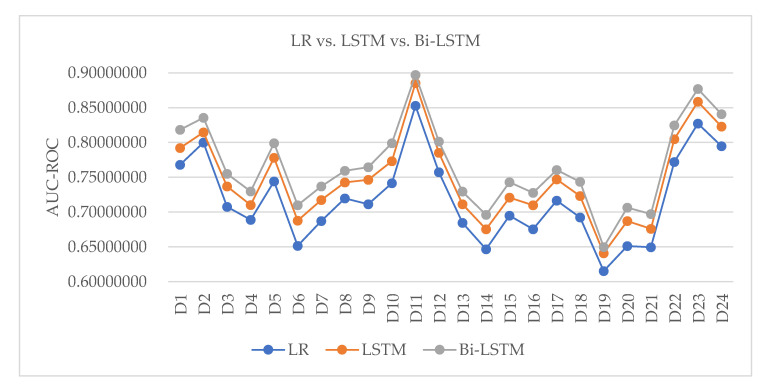
Model comparison: multinomial logistic regression (LR) vs. long short-term memory (LSTM) vs. bi-directional long short-term memory (Bi-LSTM) models.

We compared the long short-term memory (LSTM), bi-directional long short-term memory (Bi-LSTM) [15], and multinomial logistic regression (LR) models in Figure 7. The reason why we chose the multinomial logistic regression model was because, for the three models compared, the conclusions of all PVs related to diseases were consistent, but the multinomial logistic regression model required the least processing time. Thus, we selected the multinomial logistic regression model.

### 5.2. Limitations and Future Research

In this study, we demonstrated how the methods to rank PVs to support PEC can work using the MIMIC-III database. However, changes in the disease spectrum and differences between regions were not considered. In fact, for different regions, the PVs should be ranked with our method by using real clinic datasets. Then, the PV checklist should be fitted to improve the quality of PEC specific to the region. This will be the topic of our next study. Furthermore, we must try to reduce the essential PVs as much as possible for disease prediction, without the need for complicated and costly instruments. This could help provide intelligent decision support to save more lives during PEC.

## 6. Conclusions

In this paper, we proposed a decision support method for PEC based on ranking the importance of PVs. We used large-scale ICU patient data to explore the relationships between multiple PVs and specific diseases. For certain diseases, we found the importance of related PVs and determined a cohort of PVs that should be monitored in ICU patients for PEC. Considering the richness and authenticity of the data, our results are credible. Furthermore, in EMSs, this cohort of PVs will guide paramedics to care for patients more effectively. Although a specific task inspired our solution, it has the potential to be generalized to other clinical duties; for example, by switching target tags from PVs and diseases to other biomedical entities, it could be used to explore new relationships.

## Figures and Tables

**Table 1:** The 24 diseases focused on in this study.

No.	Disease Name	No.	Disease Name
D1	Acute and unspecified renal failure	D13	Disorders of lipid metabolism
D2	Acute cerebrovascular disease	D14	Essential hypertension
D3	Acute myocardial infarction	D15	Hypertension with complications and secondary hypertension
D4	Cardiac dysrhythmias	D16	Fluid and electrolyte disorders
D5	Chronic kidney disease	D17	Gastrointestinal hemorrhage
D6	Congestive heart failure; non-hypertensive	D18	Other liver diseases
D7	Chronic obstructive pulmonary disease and bronchiectasis	D19	Other lower respiratory diseases
D8	Complications of surgical procedures or medical care	D20	Other upper respiratory diseases
D9	Conduction disorder	D21	Pleurisy; pneumothorax; pulmonary collapse
D10	Coronary atherosclerosis and other heart diseases	D22	Pneumonia (except that caused by tuberculosis or sexually transmitted diseases)
D11	Diabetes mellitus with complications	D23	Respiratory failure; insufficiency; arrest (adult)
D12	Diabetes mellitus without complications	D24	Septicemia (except in labor)

**Table 2:** The 17 PVs analyzed in this study.

No.	PV Name (Abbreviation)	No.	PV Name (Abbreviation)
PV1	Oxygen saturation (SpO2)	PV10	Temperature (TEMP)
PV2	Capillary refill rate (CRR)	PV11	Systolic blood pressure (SBP)
PV3	Fraction inspired oxygen (FiO2)	PV12	Respiratory rate (RR)
PV4	Glasgow coma scale total (GCST)	PV13	Mean blood pressure (MBP)
PV5	Glasgow coma scale eye opening (GCSE)	PV14	Height (Ht)
PV6	Glasgow coma scale motor response (GCSM)	PV15	Heart rate (HR)
PV7	Glasgow coma scale verbal response (GCSV)	PV16	Glucose (Glu)
PV8	pH (PH)	PV17	Diastolic blood pressure (DBP)
PV9	Weight (Wt)		

**Table 3:** Examples of ranking the importance of PVs for different diseases.

Rank	Conduction Disorder	Coronary Atherosclerosis and Other Heart Disease	Diabetes Mellitus with Complications	Diabetes Mellitus without Complications
1	HR ^1^	HR	Glu	Glu
2	CRR	CRR	SBP	Weight
3	SpO2	SpO2	RR	SBP
4	RR	GCSM	MBP	MBP
5	GCST	Glu	HR	TEMP
6	TEMP	RR	PH	HR
7	GCSV	TEMP	Ht	GCSE
8	DBP	SBP	GCSE	SpO2
9	GCSE	MBP	GCST	CRR
10	Glu	DBP	TEMP	Ht
11	GCSM	GCST	DBP	DBP
12	PH	GCSE	GCSM	PH
13	Ht	PH	FiO2	GCST
14	SBP	GCSV	GCSV	RR
15	MBP	FiO2	Weight	GCSM
16	FiO2	Ht	SpO2	GCSV
17	Weight	Weight	CRR	FiO2

^1^ PV abbreviation of heart rate. All the PVs have been defined in Table 2.
